# High Nasal Carriage Rate of *Staphylococcus aureus* Containing Panton-Valentine leukocidin- and EDIN-Encoding Genes in Community and Hospital Settings in Burkina Faso

**DOI:** 10.3389/fmicb.2016.01406

**Published:** 2016-09-13

**Authors:** Abdoul-Salam Ouedraogo, Catherine Dunyach-Remy, Aimée Kissou, Soufiane Sanou, Armel Poda, Carole G. Kyelem, Jérôme Solassol, Anne-Laure Bañuls, Philippe Van De Perre, Rasmata Ouédraogo, Hélène Jean-Pierre, Jean-Philippe Lavigne, Sylvain Godreuil

**Affiliations:** ^1^Centre Hospitalier Universitaire Souro SanouBobo Dioulasso, Burkina Faso; ^2^Département de Bactériologie-Virologie, Centre Hospitalier Universitaire de MontpellierMontpellier, France; ^3^Université de MontpellierMontpellier, France; ^4^Institut National de la Santé et de la Recherche Médicale U1058, Infection by HIV and by Agents with Mucocutaneous Tropism: From Pathogenesis to PreventionMontpellier, France; ^5^Institut National de la Santé et de la Recherche Médicale U1047, Université de MontpellierNîmes, France; ^6^Service de Microbiologie, Centre Hospitalier Universitaire CaremeauNîmes, France; ^7^Department of Biopathology, Centre Hospitalier Universitaire MontpellierMontpellier, France; ^8^Department of Clinical Oncoproteomics, Montpellier Cancer InstituteMontpellier, France; ^9^UMR MIVEGEC (IRD 224 - Centre National de la Recherche Scientifique 5290 - Université de Montpellier)Montpellier, France

**Keywords:** carriage, EDIN, oligonucleotide array, PVL, *Staphylococcus aureus*

## Abstract

The objectives of the present study were to investigate the rate of *S*.aureus nasal carriage and molecular characteristics in hospital and community settings in Bobo Dioulasso, Burkina Faso. Nasal samples (*n* = 219) were collected from 116 healthy volunteers and 103 hospitalized patients in July and August 2014. Samples were first screened using CHROMagar Staph aureus chromogenic agar plates, and *S. aureus* strains were identified by mass spectrometry. Antibiotic susceptibility was tested using the disk diffusion method on Müller-Hinton agar. All *S. aureus* isolates were genotyped using DNA microarray. Overall, the rate of *S. aureus* nasal carriage was 32.9% (72/219) with 29% in healthy volunteers and 37% in hospital patients. Among the *S. aureus* isolates, only four methicillin-resistant *S. aureus* (MRSA) strains were identified and all in hospital patients (3.9%). The 72 *S. aureus* isolates from nasal samples belonged to 16 different clonal complexes, particularly to CC 152-MSSA (22 clones) and CC1-MSSA (nine clones). Two clones were significantly associated with community settings: CC1-MSSA and CC45-MSSA. The MRSA strains belonged to the ST88-MRSA-IV or the CC8-MRSA-V complex. A very high prevalence of toxinogenic strains 52.2% (36/69), containing Panton-Valentine leucocidin- and EDIN-encoding genes, was identified among the *S. aureus* isolates in community and hospital settings. This study provides the first characterization of *S. aureus* clones and their genetic characteristics in Burkina Faso. Altogether, it highlights the low prevalence of antimicrobial resistance, high diversity of methicillin-sensitive *S. aureus* clones and high frequency of toxinogenic *S. aureus* strains.

## Introduction

*Staphylococcus aureus* is both a human commensal and a frequent cause of clinically important infections in hospital and community settings. It colonizes about one third of healthy humans and is most often found in the nose (Kaspar et al., 2016). As pathogenic agent, *S. aureus* causes a wide range of infections (e.g., skin and soft tissue infections, sepsis, pneumonia, osteomyelitis, endocarditis; Shittu et al., 2011). Epidemiological studies of staphylococcal infections showed that nasal carriage is a risk factor and usually the origin of the infection (Wertheim et al., 2004). *S. aureus* treatment is complicated by the global spread of methicillin-resistant *S. aureus* (MRSA) strains (Holden et al., 2013).

Studies on the worldwide spread of *S. aureus* indicated that clone predominance varies according to the continent: ST300 in USA, ST59 in Asia, ST30/USA1100 in Southwest Pacific Oceania, ST93 in Queensland and ST80 in European countries (Fluit et al., 2015). In Africa, the limited epidemiological data suggest that *S. aureus* clone distribution is particularly heterogeneous, possibly due to the huge cultural and geographical diversity. Similarly, toxinogenic strains are unequally distributed in the different countries. For example, the prevalence of *S. aureus* strains that produce Panton-Valentine Leukocidin (PVL) is of 0.3% in South Africa and of 100% in Tunisia (Schaumburg et al., 2014; Abdulgader et al., 2015). Conversely, no data are available on the distribution of *S. aureus* strains that produce epidermal differentiation inhibitor (EDIN), one of the main virulence factors involved in *S. aureus* diffusion. As several pathogenic *S. aureus* clones, such as the European clone ST80-MRSA-IV (Messad et al., 2013), express EDIN or EDIN-like exotoxins, EDIN-positive strains could also be present in the African continent, especially in Maghreb where the ST80 clone is very common.

Few studies focused on *S. aureus* in hospital or community settings in Burkina Faso, a low-income country of West Africa, and nothing is known about clone circulation. The objectives of the present study were to characterize the *S. aureus* isolates in nasal specimens from hospitalized and healthy volunteers in Bobo Dioulasso, Burkina Faso.

## Materials and methods

### Patients, specimen collection, and ethical clearance

The study was approved by the local ethics committee (Souro Sanou University Hospital board; authorization number: MS/SG/CHUSS/DG/DL 2014-171, 2 July 2014). All participants signed a written informed consent for participation in the study. From 1st July to 31th August 2014, 116 randomly selected healthy volunteers from the community and 103 patients hospitalized (not for *S. aureus* infection) at the Souro Sanou University Hospital (Bobo Dioulasso, Burkina Faso) for more than 48 h were included prospectively and consecutively in the study. Souro Sanou University Hospital is the major healthcare and referral center for the southern and western regions of Burkina Faso. During this period only one nasal swab sample by individuals was collected excluding the possibility to study the dynamics of SA nasal carriage over time (distinction between intermittent and persistent carriers).

Nasal specimens from healthy volunteers and hospitalized patients were collected using double-head flocked swabs (Copan®, Italy). The following epidemiological and clinical data were recorded for all participants: age, gender, antibiotic treatment during the past 3 months and hospital stays in the previous year.

### Microbiological study of *S. aureus*

Nasal swab samples were plated on CHROMagar Staph aureus chromogenic medium (BD Diagnostics, Sparks, Md.) and examined after 24 and 48 h of incubation at 37°C, following the manufacturer's recommendations. All mauve to orange/mauve colonies were considered as positive for the presence of *S. aureus*. The identification was confirmed by matrix-assisted laser desorption ionization-time of flight (MALDI-TOF) mass spectrometry (Bruker Daltonics, Germany). A multiplex real-time PCR assay that targets bacterial *nuc* gene to distinguish *S. aureus* from other Staphylococcus spp., and the *mecA* gene for the detection of methicillin resistance was used. In all confirmed *S. aureus* isolates, antibiotic susceptibilities were then determined using the disk diffusion method on Müller–Hinton agar with the following antibiotics: amoxicillin, amoxicillin-clavulanic acid, oxacillin, gentamicin, tobramycin, tetracycline, erythromycin, lincomycin, trimethoprim/sulfamethoxazole, ofloxacin, fusidic acid, fosfomycin, and vancomycin. Results were interpreted following the European Committee on Antimicrobial Susceptibility Testing (EUCAST) clinical breakpoints (Version 5.0; http://www.eucast.org/clinical_breakpoints/). Susceptibility to methicillin was screened with the cefoxitin disk diffusion method.

### Oligonucleotide DNA arrays and genotyping

Each *S. aureus* strain was analyzed at the INSERM laboratory in Nîmes, France. The *S. aureus* genotyping kit (Alere Technologies GmbH, Germany) was used according to previously detailed protocols and procedures (Monecke et al., 2008). The DNA microarray covers 332 different target sequences, including species, capsule and *agr* group typing markers, resistance genes (SCC*mec, mecA, mecC*) and genes encoding exotoxins or Microbial Surface Components Recognizing Adhesive Matrix Molecules (MSCRAMM). The affiliation of isolates to clonal complexes (CCs) or sequence types (STs), defined based on MLST and *spa*-typing (Monecke et al., 2008), was determined by automated comparison of the hybridization profiles to a collection of previously characterized reference strains (Monecke et al., 2008, 2011).

### Statistical analysis

The nasal carriage rate and the frequency of MRSA strains were analyzed relative to the demographic and clinical characteristics of to the clonal complexes (CCs) and the expression of the different virulence genes, using the Fisher's exact test. Statistical analyses were performed using the S-Plus 2000 software package (Insightful Corporation, Seattle, WA, USA) and results were considered significant at *p* < 0.05.

## Results

### Prevalence and risk factors of *S. aureus* nasal carriage

During the study period, 219 subjects (116 healthy volunteers and 103 hospitalized patients) were enrolled. The demographic characteristics and possible risk factors for *S. aureus* colonization in the two groups are shown in Table 1. Overall, the participants' mean age was 24.1 years (±14.6) and 69.4% of them were males. Among healthy volunteers, 13 (11%) had been hospitalized in the previous year and 18 (16%) received antibiotics within the 3 months prior to inclusion in the study: fluoroquinolones (*n* = 9/18; 50%), ß-lactams (*n* = 7/18; 39%), or others (*n* = 2/18, 11%). Among the hospitalized patients, 14 (14%) had been hospitalized in the previous year. Significantly more patients than healthy volunteers had received antibiotics within the last 3 months (37 vs. 16%, *p* < 0.001). No significant difference could be observed concerning the type of antibiotic treatment in the two populations (Table 1).

**Table 1:** **Demographic characteristics, prevalence of *Staphylococcus aureus* nasal carriage and risk factors in the study population**.

**Characteristics**	**Healthy volunteers *n* = 116**	**Inpatients *n* = 103**	**Total *n* = 219**	***P* H vs. I**
Age (mean, SD), *y*	22.2 (11.7)	26.3 (17)	24.1 (14.6)	NS
Hospitalization duration (mean, SD), day	–	9.6 (4.2)	–	NA
**HOSPITAL SERVICE**				
Pediatrics, *n* (%)	–	32 (31.1)	–	NA
Surgery, *n* (%)	–	27 (26.2)	–	NA
General Medicine, *n* (%)	–	25 (24.3)	–	NA
Gynecology/obstetrics, *n* (%)	–	19 (18.4)	–	NA
*S. aureus* carriers, *n* (%)	34 (29.3)	38 (36.9%)	72 (32.9)	NS
MRSA carriers, *n* (%)	0 (0)	4 (3.9%)	4(1.82)	NS
Previous hospitalization (in the last year), *n* (%)	13 (11.2)	14 (13.6)	27 (12.3)	NS
Antibiotic treatment (in last 3 months), *n* (%)	18 (15.5)	38 (36.9)	56 (25.6)	<0.001
ß-lactams, *n* (%)	7 (6)	16 (15.55)	23 (10.5)	NS
Fluoroquinolones, *n* (%)	9 (7, 75)	13 (12.6)	22 (10)	NS
Other antibiotics^*^, *n* (%)	2 (1.75)	9 (8.75)	11 (5)	NS

* *Tetracycline, doxycycline, co-trimoxazole (trimethoprim/sulfamethoxazole), erythromycin, chloramphenicol. NS, not significant; NA, not applicable*.

*S. aureus* was identified in the nasal samples of 32.9% (72/219) participants (29.3% of healthy volunteers and 37% of hospitalized patients; *p* = 0.23). Moreover, the rate of *S. aureus* carriage varied in the different hospital services: 59% (16 of the 27 patients from this department) in the Surgery Department, 37% (12/32) in the Pediatric, 32% (8/25) in the General Medicine, and 11% (2/19) in the Gynecology/Obstetric Department. Univariate analysis did not identify any specific risk factor associated with *S. aureus* nasal carriage in the whole population and the two groups separately. Among the *S. aureus* isolates, MRSA frequency was 0 (*n* = 0) in healthy volunteers and 3.9% (*n* = 4) in hospitalized patients (*p* = 0.25), representing a total occurrence of 1, 82%.

### *S. aureus* antimicrobial susceptibility testing

**Figure 1:**
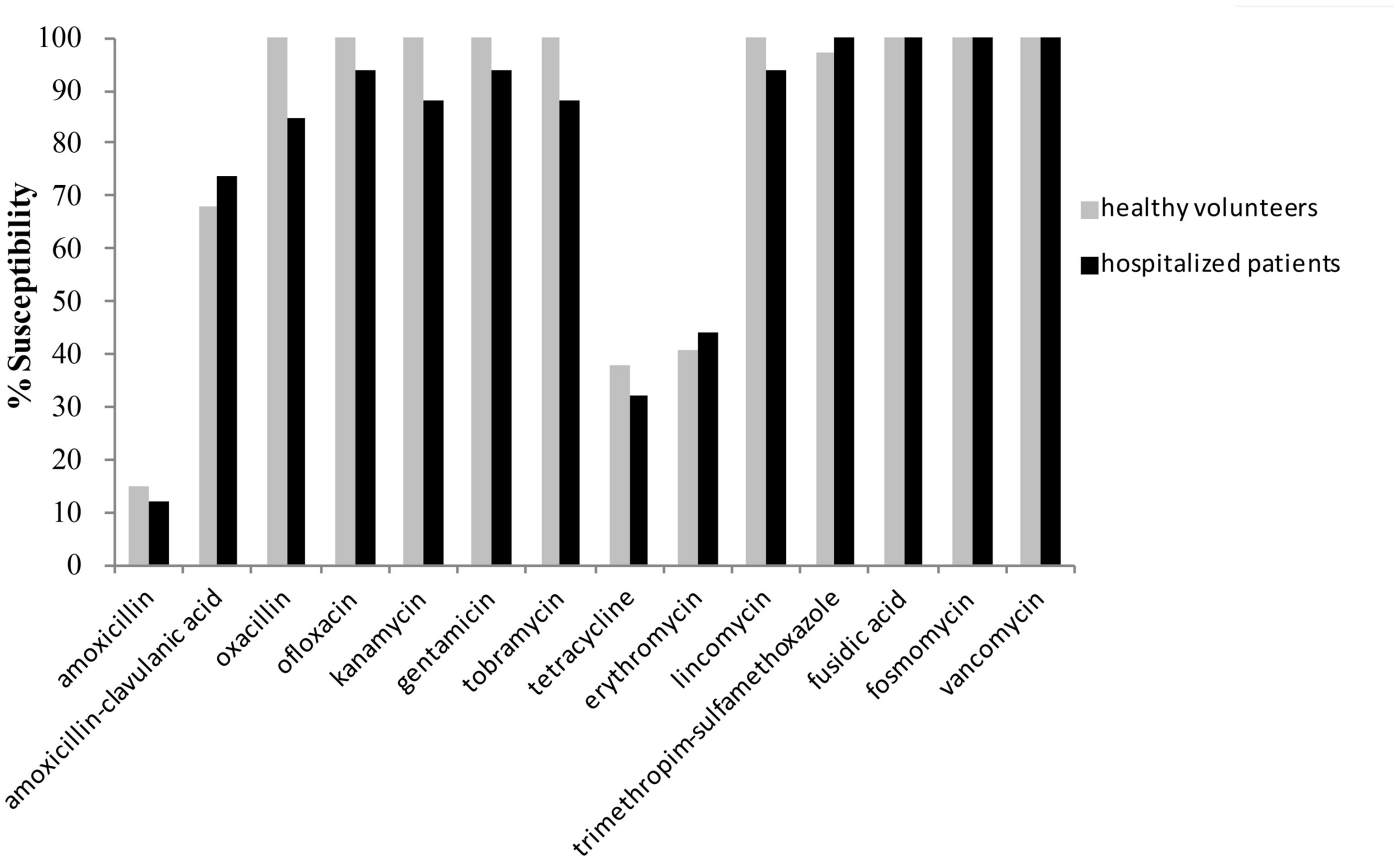
**Percentage of antimicrobial susceptibility for the *Staphylococcus aureus* strains isolated from healthy volunteers (*n* = 34) and hospitalized patients (*n* = 38) (no significant differences between groups)**.

Analysis of the *in vitro* activities of antimicrobial agents against the 72 *S. aureus* isolates highlighted a low level of resistance to all classical anti-staphylococcal treatments (Figure 1). All isolates were susceptible to vancomycin, fosfomycin, and fusidic acid. In strains isolated from hospitalized patients, the trimethoprim/sulfamethoxazole association was always active; in strains from healthy volunteers, oxacillin, ofloxacin, and aminoglycosides (kanamycin, gentamicin, and tobramycin) were always active. The highest resistance rates were observed for amoxicillin (90%) followed by tetracycline (61%) and erythromycin (54%), Single one MRSA isolate was resistant to aminoglycosides and also to fluoroquinolones.

### Distribution of *S. aureus* clonal complexes

Clonal complexes (CC) characterization in the 72 isolates allowed comparing the *S. aureus* populations (diversity and origin) of hospitalized patients and healthy volunteers and highlighted the great diversity of *S. aureus* strains circulating in the studied region (Table 2). ST 152-MSSA (*n* = 15) and CC1-MSSA (*n* = 8) were the main clones isolated in hospitalized patients and healthy volunteers, respectively. Overall, methicillin-susceptible *S. aureus* (MSSA) strains belonged to many different CCs: ST 152 (*n* = 22), CC5 (*n* = 14), CC1 (*n* = 9), ST 121 (*n* = 5), CC45 (*n* = 5), ST 707 (*n* = 2), ST 15 (*n* = 2), CC22 (*n* = 1), CC30 (*n* = 1), ST5 (*n* = 1), ST 30 (*n* = 1), CC9 (*n* = 1), ST 152 (*n* = 1). The Four MRSA strains belonged either to the CC88-MRSA-IV or to the CC8-MRSA-V lineage (no significant difference. Three isolates were classified in unknown CCs.

**Table 2:** **Clonal complex distribution of the *Staphylococcus aureus* strains isolated from nasal samples of hospital patients and community volunteers in Burkina Faso**.

	**Hospital *n* (%)**	**Community *n* (%)**	**Total *n* (%)**
ST152-MSSA (PVL+)	15 (41.7)	7 (21.2)	22 (30.6)
CC5-MSSA	8 (22.1)	6 (18.2)	14 (19.4)
CC1-MSSA	1 (2.8)	8 (24.2)	9 (12.5)
ST121-MSSA (PVL+)	3 (8.2)	2 (6.1)	5 (6.9)
CC45-MSSA	0 (0)	5 (15.2)	5 (6.9)
CC88-MRSA	2 (5.6)	0 (0)	2 (2.8)
ST707-MSSA	2 (5.6)	0 (0)	2 (2.8)
CC8-MRSA	2 (5.6)	0 (0)	2 (2.8)
ST15-MSSA (PVL+)	0 (0)	2 (6.1)	2 (2.8)
CC22-MSSA	1 (2.8)	0 (0)	1 (1.4)
CC30-MSSA	1 (2.8)	0 (0)	1 (1.4)
ST5-MSSA (PVL+)	1 (2.8)	0 (0)	1 (1.4)
ST30-MSSA (PVL+)	0 (0)	1 (3.0)	1 (1.4)
CC9-MSSA	0 (0)	1 (3.0)	1 (1.4)
CC152-MSSA	0 (0)	1 (3.0)	1 (1.4)
None	2 (5.6)	1 (3.0)	3 (4.1)
TOTAL	38 (100)	34 (100)	72 (100)

### Antibiotic resistance and virulence gene profiles

The antibiotic resistance gene profiles obtained by DNA array analysis were in full agreement with the data obtained by conventional susceptibility testing. All MRSA isolates detected with the cefoxitin disk diffusion test carried the *mecA* gene within the SCC*mec* mobile genetic element. *MecC* was not detected in any of our samples. The most prevalent macrolide resistance gene was *ermC* (25 isolates, 36.2%), whereas *ermA* was never found. Only one aminoglycoside resistance gene (*aacA-aphD*) was detected and only in one MRSA strain, confirming the still high antimicrobial activity of this antibiotic family. The *tetK* efflux gene (tetracycline resistance) was detected in 36 isolates (52.2%). No *van* gene was detected in agreement with the *in vitro* susceptibility data.

Some distinctive features regarding the virulence factor profiles were observed. Between 14 and 42% of strains expressed enterotoxin-coding genes [*seg* (*n* = 29, 42%), *sek* and *seq* (*n* = 12, 17.4% for each), and *sea* (*n* = 10, 14.5%)]. Conversely, genes encoding haemolysins (*hlg, hlgv, hlgA, hld*), intracellular adhesion proteins (*icaA, icaC, icaD*) or MSCRAMMs (*ebpS, clfA, clfB, fnbA, fnbB*, and *bbp*) were detected in more than half and often all strains. The *agr1* allele was found in 42% of isolates (*n* = 29). Toxinogenic genes were frequently present in our strains. Although *eta* and *etb* (the genes encoding exfoliatin A and B) were never detected, 44.9% (*n* = 31) and 29% (*n* = 20) of isolates expressed the genes that encode PVL (*lukS-PV/lukF*-PV) and toxic shock syndrome toxin 1 (TSST-1; *tst)*, respectively. Moreover, the prevalence of *edin*-positive isolates was 46.4% (*n* = 32) and specifically, 14.5% (*n* = 10) of all isolates were *edinA*-positive and 31.9% (*n* = 22) *edin-B* positive. The *edin-B* and *etD* genes were always co-expressed.

### Main characteristics of the *edin*+ and PVL+ *S. aureus* isolates

The *edin-B*+ strains (*n* = 22) belonged to the ST152 clone (*spa* type t355). In this clone, *edin-B, etD*, and *lukF/lukS-PV* genes are associated. All 22 strains expressed the *sarA* regulon that controls *edin-B* expression, and belonged to the *agr*I cluster. No enterotoxin gene was detected. However, all *edin-B*+ strains expressed one or more genes encoding haemolysins (*hlgv, hlgA hla*, and *hlb*), intracellular adhesion proteins (*icaA, icaD*), type 5 capsular polysaccharides (*cap5)*, immune evasion proteins (*sak, scn*), proteases (*sspA, sspB*), some MSCRAMMs (*bbp, clfA, clfB, cna, ebpS, fnbA, fnbB, sdrD*), defensin (*mprF*), and hyaluronidases (*hysA1, hysA2*).

The *edin-A*+ strains (*n* = 10) belonged to the clonal complexes ST73 (CC5-MSSA, *n* = 8), ST5 (*n* = 1), and ST707 (*n* = 1). The ST5 strain co-expressed *edin-A* and *lukF/lukS-PV*. All carried the *sak, chp, scn hl, hlgA, hlgv*, and *hlA lukDE* genes. The strains belonging to the ST73 and ST5 clones expressed many virulence genes (e.g., the *egc* cluster*, agrII, cap5, bbp, clfA, clfB, ebpS, fib, fnbA*, and *fnbB)*. Finally, the ST707 strain harbored distinctive virulence traits (*sek, seq, agrIII, fnbB* alone, *sdrC* and *sdrD*).

Concerning the PVL+ strains (*n* = 31), they belonged to the ST152 (*n* = 23), ST121 (*n* = 4), ST15 (*n* = 2), ST30 (*n* = 1), and ST5 (*n* = 1) clones. They all expressed *agrI* and *cap5*, with the exception of the ST121 strains that carried *agrIV* and *cap8*. Moreover, all PVL+ strains expressed genes encoding haemolysins (*hlgv, hlgA hla*), immune evasion proteins (*sak, scn*) and MSCRAMMs (*bbp, clfA, clfB, ebpS, fnbA, fnbB*).

## Discussion

This study provides the first characterization (clones and genetic traits) of *S. aureus* samples isolated from nasal specimens in Burkina Faso. Our findings highlight the low prevalence of antimicrobial resistance, the high diversity of MSSA clones and the high frequency of toxinogenic isolates among the *S. aureus* strains that circulate in community and hospital settings.

In Africa, *S. aureus* epidemiological data remain scarce. The distribution of MRSA clones is relatively heterogeneous on this continent with CC5 as the predominant clonal complex in healthcare settings. The ≪Brazilian/Hungarian≫ clone (hospital-associated MRSA ST239/ST241-III) has been identified in Maghreb, West Africa, and South African countries (Schaumburg et al., 2014; Fluit et al., 2015). The European ST80-IV clone is limited to Algeria, Egypt, and Tunisia (Schaumburg et al., 2014; Fluit et al., 2015). The community clones ST8-IV and ST88-IV have been reported both in hospital and community settings in West Africa. The ST88-MRSA-IV was also found in our population associated with the ST8-MRSA-V clone (Abdulgader et al., 2015). However, the percentage of MRSA strains (2.3%) was very low in our panel, as previously reported in Gabon (3%) (Kraef et al., 2015). On the other hand (and this is the first major finding of this study), our data highlight the high prevalence of strains harboring PVL-encoding genes (45%). These genes encode a bi-component cytotoxin that binds to the complement receptor C5a of neutrophils and that leads to severe tissue necrosis due to its cytotoxic effect on granulocytes (Dufour et al., 2002; Shallcross et al., 2013). Sub-Saharan Africa is characterized by a high dissemination of PVL+ *S. aureus* strains that mainly belong to three major clones: ST152, ST121, and ST15 (Rasigade et al., 2010; Schaumburg et al., 2014). Our study confirms this trend. The ST152 clone seems to be frequent and widespread in West Africa (40–60% of all strains) both in the community and in hospital settings (Ruimy et al., 2008; Shittu et al., 2012; Kraef et al., 2015). This clone has also been detected in Europe (Jappe et al., 2008; Perez-Roth et al., 2010; Krziwanek et al., 2011), Turkey (Sudagidan and Aydin, 2010), and Haiti (Rosenthal et al., 2014).

The high proportion of *edin*-positive MSSA (46%) strains in our population is the second main finding of this study. Indeed, such high prevalence has never been described before (14% in a previous study) (Munro et al., 2011). Three *edin* alleles have been characterized (Sugai et al., 1992; Yamaguchi et al., 2001, 2002). They all share similar biochemical activities toward Rho proteins, but are characterized by different genetic backgrounds. For instance, the *edin-A* gene is located on a plasmid, like *edin-C*, and is closely associated with the gene encoding exfoliative toxin B (Yamaguchi et al., 2001). EDIN proteins promote *S. aureus* invasiveness, by favoring its translocation to the bloodstream (Munro et al., 2010; Messad et al., 2013; Courjon et al., 2015). In our population, *edin-B* was the most prevalent *edin* gene associated with *S. aureus* nasal carriage. Many different *S. aureus* clones harbor these genes, demonstrating the wide geographical distribution of EDINs. In our population, *edinB* was more frequently detected in isolates belonging to the ST152 clone, like for the PVL+ strains. Therefore, our data indicate that strains belonging to this clone can co-express *edinB*/*etD*/*lukSF-PV*, which are the prevalent virulence markers detected in infectious strains. More worrying, in Nigeria, the ST152 clone has been associated with the *SCCm*ec cassette (Shittu et al., 2012). These findings suggest that these strains could pose serious problems in the future, as potential reservoirs for resistance and virulence factors, and they could lead to the emergence and diffusion of PVL+ MRSA clones in Burkina Faso, with a risk of severe infections, as recently discussed (Vignaroli et al., 2016).

Nasal colonization often precedes clinical infection, frequently caused by the same isolate (von Eiff et al., 2001). Therefore, nasal carriage rates and the molecular characterization of the colonizing isolates provide important insights into the risk of *S. aureus* infection in Burkina Faso. The high proportion of EDIN+ and PVL+ *S. aureus* observed in this study indicates that conditions that increase the risk of inter-individual transmission (e.g., skin-to-skin and skin-to-fomite contacts) could represent important spreading routes in Burkina Faso. One the principal limitation of this study was the absence of distinction between intermittent and persistent carriers. It is now well-established that this distinction is important as persistent carriers are at a higher risk of developing active auto-infections than intermittent and non-carriers (Wertheim et al., 2005; Muthukrishnan et al., 2013). A larger sample of strains to study the dynamics of SA nasal carriage over time in Burkina would provide a more accurate measure of risk factors for infection according to patient status (intermittent and persistent carriers).

Classically, mortality associated with severe *S. aureus* infections is particularly high in emerging countries. Recent studies have identified *S. aureus* as the main etiological agent of many infections in Sub-Saharan Africa (Shittu et al., 2011). Microbiologists and clinicians should be aware of the threat represented by the ST152 clone and implement the necessary control measures to prevent its spread.

## Author contributions

Conceived and designed the experiments: AO, CD, HJ, PV, AB, JS, RO, JL, and SG. Performed the experiments: AO, CD, AK, SS, AP, and CK. Contributed reagents/materials/analysis tools: AO, AP, CD, AK, JL. Contributed to the writing of the manuscript: AO, HJ, AB, JS, RO, JL, and SG.

### Conflict of interest statement

The authors declare that the research was conducted in the absence of any commercial or financial relationships that could be construed as a potential conflict of interest.
